# Treatment Fidelity of a Nurse-Led Motivational Interviewing-Based Pre-Treatment in Pain Rehabilitation

**DOI:** 10.1007/s11414-015-9485-4

**Published:** 2015-12-22

**Authors:** Vera-Christina Mertens, Lars Forsberg, Jeanine A. Verbunt, Rob E. J. M. Smeets, Mariëlle E. J. B. Goossens

**Affiliations:** Research Unit INSIDE, Institute for Health and Behaviour, University of Luxembourg, 11, Porte des Science, L-4366 Esch-sur-Alzette, Luxemburg; Department of Rehabilitation Medicine, Maastricht University, School for Public Health and Primary Care (CAPHRI), Postbus 616, 6200 MD Maastricht, The Netherlands; Department of Clinical Neuroscience, Karolinska Institute, Liljeholmstorg 7B, plan 6, 11726 Stockholm, Sweden; Adelante Centre of Expertise in Rehabilitation and Audiology, Postbus 88, 6430 AB Hoensbroek, The Netherlands; Department of Rehabilitation Medicine, Maastricht University Medical Center (MUMC+), Postbus 5800, 6202 AZ Maastricht, The Netherlands; Faculty of Psychology and Neurosciences (FPN), Department of Clinical Psychological Sciences (CPS), Maastricht University, Postbus 616, 6200 MD Maastricht, The Netherlands

## Abstract

Treatment fidelity and proficiency of a nurse-led motivational interviewing (MI)-based pre-treatment and control condition was evaluated. A random sample was scored by means of the Motivational Interviewing Treatment Integrity (MITI) scale, and a second rater was in charge. MI fidelity was satisfactory for three out of five ratings. Most mean ratings were higher in the MI-based intervention, but differences were not statistically significant. The threshold for beginning MI proficiency was only exceeded for one score and one additional measure. In general, higher levels of fidelity in the intervention condition confirmed that MI was partially applied there. Although the quality of MI delivery as well as mixed inter-rater reliabilities of the fidelity scores leaves room for improvement, robust findings between the two raters were found. These results suggest the need for rigor selection of MI counselors on beforehand, and continuous supervision. Furthermore, fidelity check in studies using MI is needed.

## Introduction

Motivational interviewing (MI) is a person-centered form of counseling to elicit and strengthen motivation for change.^1^ Motivation and adherence challenges are not unique to addiction treatment, wherein the historical roots of MI lay, but MI is also promising for other applications such as to promote treatment adherence.^2^ Several systematic reviews and meta-analyses showed the effectiveness of Motivational interviewing across behaviors and contexts in health care.^3^^–^6 The effect of MI as pre-treatment has specifically been acknowledged.^3^^,^^4^^,^^7^ Positive effects in pain rehabilitation treatment^8^^–^11 and moderate quality evidence have been provided for its successful application to promote physical activity in people with chronic health conditions.^12^

MI has two components: (1) the relational component consists of the so-called MI spirit, a counselor-attitude characterized by genuine interest in the client and empathy; (2) the technical component consists of techniques to evocate, elicit, and reinforce change talk^13^ (by, e.g., the use of open questions and reflections). However, so far, MI has mostly been emphasized as a spirit rather than a technique.^14^ At first sight, MI principles—asking open questions, giving reflections—look simple. But, the underlying principles resulting in MI spirit like empathy are a complex mix of skills that take considerable time to learn.^1^ As a consequence, the quality of MI delivery can vary tremendously. Since the quality of MI delivered is an important factor for the beneficial effect, this diversity can have a huge impact.^15^ For this reason, it is very important to check and accurately test whether MI is delivered as intended,^16^^,^^17^ or, in other words, to check its treatment fidelity (sometimes referred to as treatment integrity).

This can have important implications for the conclusion drawn regarding effectiveness. For example, results could be related to something else than the hypothesized working mechanism of the MI intervention because of non-adherence to procedures or failing therapist competence.

From a few other studies, it is known that MI fidelity measures have predictive validity to predict patient behavior following MI treatment.^18^^–^20 Additionally, in terms of therapists’ competence, MI training is related to MI fidelity by suppressing MI countering responses related to resistance and poorer outcome which has been linked to increased change talk which in turn predicts behavior change in MI.^20^

As MI practitioners seem to overestimate their functioning, self-report of MI fidelity can be seen as unreliable.^21^^,^^22^ Furthermore, fidelity measures can serve as manipulation check to discriminate MI reliably from non-MI-based control interventions.^23^ Thus, quality assurance based upon recordings of the actual sessions and the usage of MI-specific coding instruments can estimate whether MI was actually delivered.

However, regardless this hypothesized impact of variety in MI quality delivered, only 17% of research specifically within the field of MI research assessed fidelity adequately.^5^ In the domain somatoform disorders and of research in chronic pain specifically, two of the four studies using MI checked for treatment fidelity^8^^,^^9^ and two other studies did not.^10^^,^^11^

The aim of the present study is to evaluate the treatment fidelity of an MI-based intervention and an educational control treatment in pain rehabilitation. It is hypothesized that MI proficiency will be higher in the intervention condition and that the intervention condition can be distinguished from a non-MI-based educational control condition.

## Methods

This study is part of a large two-armed randomized controlled trial (RCT) (a detailed description of the RCT is given in detail elsewhere).^24^ This study assessed the effectiveness of a nurse-led MI-based pre-treatment compared to an attention-control pre-treatment pain education in a Dutch chronic musculoskeletal pain population before the start of the actual pain rehabilitation treatment.

In Table 1, an overview of the content of both study conditions is given.^24^

**Table 2:** Comparison of the means for the MITI global scores in educational control condition and MI-based intervention condition (*n* = 64)

Criterion	Mean control condition (*n* = 27)	Mean intervention condition (*n* = 37)	MITI 3.1.1 threshold (beginning proficiency)	Threshold exceeded?	Mann-Whitney *U*	*z*	*p* (two-tailed)
Global counselor ratings (range 1–5)							
Evocation	1.65	3.62	3.5	+	421.00	−0.93	0.34
Collaboration	1.59	3.43	3.5	–	438.50	−0.87	0.39
Autonomy/support	2.85	3.19	3.5	–	418.50	−0.17	0.10
Direction	4.58	4.49	3.5	++	404.50	−1.38	0.18
Empathy	2.67	3.95	3.5	+	410.00	−1.12	0.18
Behavior counts							
# Giving information^a^	17.81	16.86	–	n/a	−0.447	62	0.65
# MI adherent responses	1.15	0.59	–	n/a	446.00	−0.79	0.43
# MI non-adherent responses	2.37	1.24	–	n/a	471.00	−0.40	0.69
# Closed questions^a^	5.56	13.89	–	n/a	6.34	62	0.00
# Open questions	2.30	10.62	–	n/a	488.00	−0.16	0.87
Total questions^a^	7.85	24.51	–	n/a	7.99	62	0.00
# Simple reflections^a^	7.78	16.89	–	n/a	6.50	55.27	0.00
# Complex reflections^a^	2.93	4.84	–	n/a	2.51	62	0.01
Total reflections^a^	10.70	21.73	–	n/a	5.89	62	0.00
Summary scores and belonging thresholds							
Spirit	2.01	3.41	3.5	–	394.50	−1.29	0.20
% Open questions	35.66	40.09	50	–	447.00	−0.54	0.59
% Complex reflections^a^	23.01	22.72	40	–	−0.17	44.49	0.86
Reflections-to-questions ratio	3.53	1.01	1	++	464.50	−0.30	0.76
% MI adherent responses	33.70	37.65	90	–	223.50	−0.75	0.45
Two additional MI fidelity measures and belonging thresholds							
Empathy	2.67	3.95	3.5	+	410.00	−1.12	0.18
# MI non-adherent responses	2.37	1.24	–	+	471.00	−0.40	0.69

*+* threshold exceeded for one condition, *++* threshold exceeded for both conditions

^a^For those ratings, assumptions for parametric testing (independent *t* test) are fulfilled; therefore, mean, *t*, and *df* are presented in the subsequent cells

### Training of the nurses

Nurses provided one treatment condition only and were trained specifically for the intervention they had to deliver.

Training for the intervention (motivational interviewing based pre-treatment (MIP)): in the MIP condition, the nurses were both experienced MI coaches. In two half-day sessions, the nurses’ MI knowledge and experience in the context of chronic pain rehabilitation was updated based on an evidence-based MI training tailored to their specific needs. The training was provided by a certified MI trainer. Follow-up training during the trial consisted of regular supervision (three half-days during the trial period of 1, 5 years). The training was based upon actual cases and by providing direct feedback on audio taped MIP sessions by the same MI trainer.

#### Training for the Control Condition

The two nurses of the pain education control condition were experienced in the field of (pain) rehabilitation and received a 3-h refresher training in communication skills and general principles of health education. In addition, the content of relevant chapters of the book “Mastering pain” was discussed. Follow-up training included two sessions lasting 2 h in which problems encountered were discussed.

### Measurement instrument: MITI and procedures of scoring

**Figure 1:**
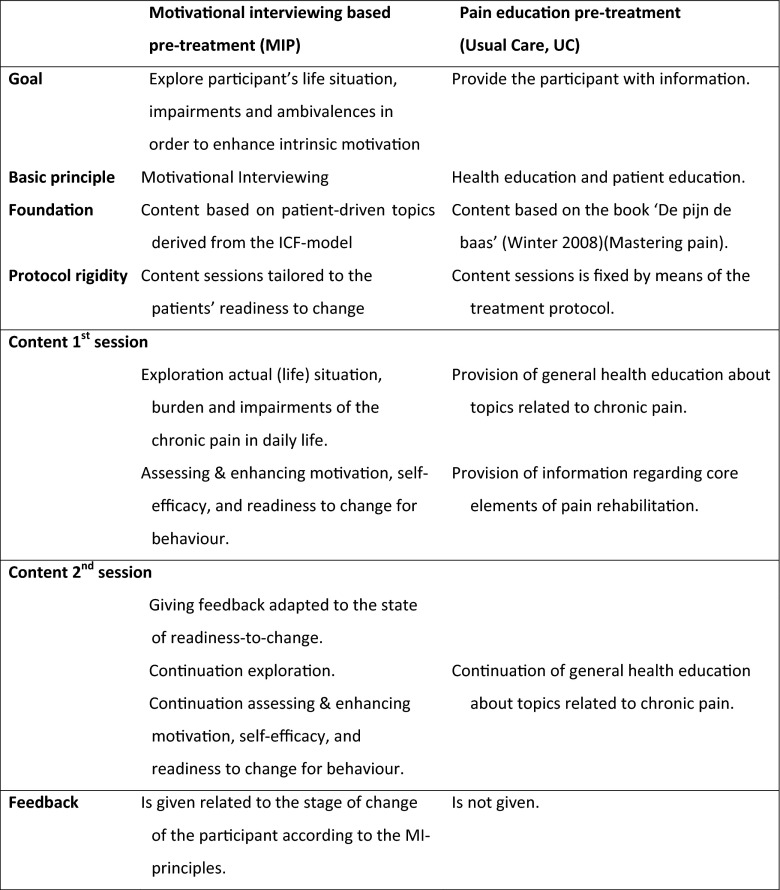
The MITI coding form

To test the treatment integrity the Motivational Interviewing Treatment Integrity (MITI, version 3.1.1) scale was used.^25^ This scale has shown to be reliable^23^^,^^26^^–^28 and valid.^25^^,^^28^ The MITI focuses exclusively on therapist’s functioning (Fig. 1).^23^

The MITI assessment instrument is composed of two different parts: “global counselor ratings” and “behavior counts” (see Fig. 1). Both were evaluated and rated during the preselected 20-min-long session sample in two separated rounds. Coding in the first round was performed without interruption. In the second round, each utterance was categorized in one of the five counselor-related behavior counts, and the total frequency of each specific behavior such as “giving information” was counted.^28^ After those two rounds of rating, five summary scores were calculated and compared to existing thresholds to evaluate fidelity finally (see end of this section).

### First round: global counselor ratings

The MITI’s global counselor ratings were designed to capture the rater’s overall impression of the session and cover five aspects: (1) evocation, (2) collaboration, (3) autonomy/support, (4) direction, and (5) empathy. A five-point scale ranging from 1 (low) to 5 (high) has to be scored by the assessor. A precise definition of each variable can be found in the MITI’s manual.^25^

### Second round: behavior counts

Next, the assessor counted the total frequency of five categories of verbal behavior: (1) giving information, (2) MI adherent responses (i.e., asking permission, emphasizing control, affirming, or supporting), (3) MI non-adherent responses (i.e., advising without permission, confronting, or directing), (4) question (open vs. closed), and (5) reflection (simple vs. complex).^25^

### MITI summary scores and belonging thresholds for beginning proficiency

After the two rounds, five indices (MITI summary scores) were calculated to evaluate MI fidelity. One is gained from the global counselor ratings: MI spirit. Four were gained from the behavior counts: (1) percentage of open questions, (2) percentage of complex reflections, (3) percentage of MI adherent responses, and (4) a reflections-to-questions ratio.

After this, the summary scores were checked against the thresholds score for “beginning proficiency” level.^25^ This threshold was defined as follows: “beginning proficiency” needs at least 3.5 points (out of 5) for the global score spirit, and a “reflection to question ratio” of 1. The “percentage of open questions” and “complex reflections” had to be at least 50 and 40%, respectively. And finally, the “percentage of MI adherent behaviors” had to be 90%.

### Two additional MI fidelity measures and belonging thresholds

Furthermore, as empathy and MI non-adherent behaviors are specifically mentioned as predictors for successful treatment,^20^^,^^29^ both were considered of such importance that they were also taken into account in the evaluation of MI fidelity. This was done by checking whether empathy crossed the threshold of the other global counselor ratings (namely 3.5), and counting the amount of MI non-adherent responses (being as low as possible as avoiding MI non-adherent responses might be more important than using MI adherent responses).^25^

### Procedures of the sampling

All sessions (intervention and control condition) were audio taped, and a random sample of 20% (*n* = 64) was used to test treatment fidelity. To collect this sample, randomization of audio taped samples was stratified for the first and second sessions, as well as for intervention and control condition. In case the actual audiotape was not available due to non-consent of the participant to record (*n* = 4) or technical problems (*n* = 12), the consecutive tape of the next participant was chosen. A research assistant selected the 20-min session.

All the selected samples were scored by the first rater (VCM), and half of these recordings (*n* = 32) were also scored by a second rater (JJ) blinded for group allocation.

### Ethics

The study was approved by the Medical Ethical the University Hospital Maastricht and Maastricht University. The study is registered in a public trial registry (Nederlands Trial Register NTR 3065). All participants provided written informed consent for as well study participation as well as audio recording of the sessions.

### Training of the MITI raters

Before the start of the study, both raters received initially a 40-h training program in MITI coding according to Moyers et al.^25^ Furthermore, both raters were re-trained at the start of the coding work by using English-spoken training materials from the Center for Alcoholism, Substance Abuse and Addictions (CASAA, University of New Mexico), and worked under supervision of an employee of one of the existing MI coding labs, MIC lab, Karolinska Institutet, Stockholm, Sweden.

Furthermore, the first six double-coded sessions served as training material. Reliability of both raters was stated two times: (1) before the start of the actual rating by calculating ICCs between the two raters and the coding lab’s rating who served as “gold standard” on similar English-spoken training materials; (2) during the actual rating, reliability was stated also (see section Statistical analysis).

During the period of rating, the first rater (VCM) participated in weekly intervision of the coding lab and could furthermore consult experienced raters. Thirty-two sessions (19 intervention conditions, 13 control conditions) were independently scored by the second rater (JJ).

### Scoring double-coded sessions

For the double-coded sessions, the raters scored the sessions independently and also had to reach consensus for the global counselor ratings afterward. To score the behavior counts, the arithmetic mean was calculated. In the following, this is referred to as consensus approach.

### Statistical analysis

To evaluate MI quality in both study conditions, scores on all MITI domains were interpreted according the manual and belonging thresholds,^25^ and the two additional MI fidelity measures.

To test for differences in MI fidelity scores between conditions, an independent *t* test (significance level of 0.05) was used. In case of non-normal distribution of either sample of the first or the second rater, the Mann–Whitney test was used.

Inter-rater reliability was calculated based on the intraclass correlation coefficients (ICCs)^30^ by means of a two-way mixed effects ICC model (absolute agreement) and interpreting single measures in the SPSS output. Inter-rater reliability was classified according Cicchetti and Sparrow (1981) who indicated ICC <0.40 as poor, 0.40–0.59 as fair, 0.60–0.74 as good, and 0.75–1.00 as excellent.^30^ Furthermore, in case of an ordinal scale (present the global counselor ratings) Krippendorff’s alpha (KALPHA)^31^ was also calculated by using a macro.^32^

Post hoc comparisons took place on nurse level in order to get more insights in nurse-specific fidelity.

Data were analyzed using Statistical Software Package for Social Sciences (SPSS), version 21 (SPSS Inc., Chicago, IL).

### Reliability/sensitivity analysis

Sensitivity analysis consisted of two subsequent steps: 1) Checking inter-rater reliability between the results of the double-coded sessions and, 2) comparing the results of two approaches of the ratings: Ratings of the first rater with the second rater as well as a consensus approach between the two raters.

## Results

The four nurses of both conditions participating in this study were experienced in the working field of (pain) rehabilitation. The two nurses of the MI-based intervention condition had 4 years of experience with MI; the two nurses of the educational control condition had experience with patient education in rehabilitation care.

A random sample *n* = 64 of all nurse-led sessions (*n* = 37 intervention conditions, *n* = 27 control conditions) was scored by the first rater (VCM). Out of this sample, *n* = 26 sessions (18 intervention conditions, 8 control conditions) were also scored by a second rater (JJ). Six sessions were used as training material at the start of the double coding.

## Overall Results

### Global counselor ratings

According to the MITI, the nurses’ beginning proficiency competence in the use of MI was satisfactory for the global counselor ratings direction (intervention as well as control condition), and empathy and evocation (intervention condition only).

Table 2 shows that all mean global counselor ratings were, except for direction (0.18), higher (between 0.34 and 1.97 points higher on a five-point Likert scale) in the MI-based intervention condition compared to the education control condition. However, mean differences were not statistically significantly different.

**Table 1:** Main features of the two interventions

	Motivational interviewing based pre-treatment (MIP)	Pain education pre-treatment (Usual Care, UC)
**Goal**	Explore participant’s life situation, impairments and ambivalences in order to enhance intrinsic motivation	Provide the participant with information.
**Basic principle**	Motivational Interviewing	Health education and patient education.
**Foundation**	Content based on patient-driven topics derived from the ICF-model	Content based on the book ‘De pijn de baas’ (Mastering pain).
**Protocol rigidity**	Content sessions tailored to the patients’ readiness to change	Content sessions is fixed by means of the treatment protocol.
**Content 1** ^**st**^ **session**		
	Exploration actual (life) situation, burden and impairments of the chronic pain in daily life. Assessing & enhancing motivation, self-efficacy, and readiness to change for behaviour.	Provision of general health education about topics related to chronic pain. Provision of information regarding core elements of pain rehabilitation.
**Content 2** ^**nd**^ **session**		
	Giving feedback adapted to the state of readiness-to-change. Continuation exploration. Continuation assessing & enhancing motivation, self-efficacy, and readiness to change for behaviour.	Continuation of general health education about topics related to chronic pain.
**Feedback**	Is given related to the stage of change of the participant according to the MI-principles.	Is not given.

### Behavior counts

The MI-based intervention condition had statistically significant higher scores for the amount of closed questions, total questions, simple reflections, complex reflections, and the amount of total reflections.

### Summary scores and belonging thresholds of beginning proficiency

In terms of MI competence according to the MITI, the threshold for beginning proficiency was exceeded for the reflections-to-questions ratio only (intervention as well as control condition).

The percentage of open questions and the percentage of MI adherent responses showed slightly higher mean ratings in the intervention condition compared to the control condition (40.09 and 35.66%, respectively, by a threshold of 50%, and 37.65 and 33.70%, respectively by a threshold of 90%).

The percentage of complex reflections (23.01 and 22.72%, respectively, by a threshold of 40%) as well as reflections-to-questions ratio (3.53 and 1.01, respectively) were higher in the control condition compared to the MI-based intervention condition.

### Two additional MI fidelity measures and belonging thresholds

Empathy was scored higher, but not statistically different in the MI-based intervention condition, and the threshold for beginning competence was reached for the MI-based intervention.

One time less MI non-adherent responses in the intervention condition compared to the control condition were found (2.37 and 1.24, respectively).

### Reliability/sensitivity analysis

Quality of reliability between the two raters was mixed (see Table 3), ranging from poor up to excellent.

The ratings of the second rater as well as the consensus approach of both raters together confirmed the previously mentioned findings of the first rater. This led to the overall conclusion that all ratings for global counselor ratings, and five out of the seven behavior count (sub)scores were in terms of MI fidelity higher in the intervention condition compared to the control condition. Similarly, it was found that not all thresholds for beginning MI proficiency were exceeded. Only in the consensus approach, a statically significant difference for the global counselor rating evocation (<0.01) between the intervention and control condition was found. However, in no condition, the score did exceed the threshold in ratings of both raters.

In the consensus approach, intervention condition and control condition can be clearly discriminated for global scores spirit (<0.01) and collaboration (<0.02). Furthermore, the behavior counts closed questions (<0.01), total questions (<0.01), simple reflections (<0.01), complex reflections (<0.01), and total reflections (<0.01) were also discriminative.

### Nurse-specific fidelity

In the post hoc analysis, a considerable variation in nurses’ MI fidelity of the different MITI aspects within the intervention condition and control condition was found.

The nurses’ individual behavior influenced the overall ratings of MI fidelity: one nurse of the control condition scored high on the reflections-to-questions ratio explaining the higher—and threshold crossing—reflections-to-questions ratio in the control condition (see Table 2).

**Table 3:** Reliability MITI scoring for the two raters (*n* = 26)

Global counselor ratings MITI	KALPHA ordinal
Direction	0.63
Empathy	0.55
Spirit	0.62
Evocation	0.58
Collaboration	0.57
Autonomy/support	0.17
Behavior counts MITI	ICC
# Giving information	0.45*
% Open questions	0.89**
# Closed questions	0.89**
# Open questions	0.95**
# Total questions	0.96**
% Complex reflections	0.31
# Simple reflections	0.84**
# Complex reflections	0.37*
# Total reflections	0.79**
Reflections-to-questions ratio	0.94**
% MI adherent responses	0.24
# MI adherent responses	0.12
# MI non-adherent responses	0.41*

*ICC* intraclass correlation coefficient, single measures, *KALPHA* Krippendorff’s alpha

*p < 0.05; **p < 0.01

The fact that the nurses of the intervention condition used twice as many reflections as the nurses of the control condition four times as many open questions indicated that the intervention nurses used more MI required behaviors which means that MI took place in the intervention condition. This was hidden by just looking on the summary scores and belonging thresholds.

Within the intervention group, one of the two nurses of the intervention group scored higher and crossed the threshold proficiency for two additional global counselor ratings, but did not cross another threshold of the other summary scores.

## Discussion

The higher MITI mean ratings for treatment proficiency and treatment fidelity in four out of five global counselor ratings, six out of nine behavior counts, threes out of five summary scores, and two out of two additional fidelity measures confirmed that motivational interviewing was applied in the MI-based intervention condition of the underlying trial compared to its educational control condition.

MI proficiency, referred to by crossing belonging thresholds, was not present for all available domains. Furthermore, on the basis of several available domains, a statically significantly discrimination between both conditions could not be achieved. Thus, mean MI proficiency scores were higher in the MI-based intervention condition, but the levels are such that it can be debated whether the MI was delivered in such a way that it really influenced the patients’ behavior. As such, there is concern whether the MI-based intervention is not advanced enough to make a change in the outcome of the pain rehabilitation treatment.

Sensitivity analysis by taking into account different ratings approaches (first rater, second rater, consensus approach of both raters together) seems to confirm the robustness of the findings, with the remark that these findings have to be seen in the light of rather mixed reliability. Findings of poor reliability were especially present in ratings, which did not exceed the threshold. For this reason, it cannot be concluded whether this is cause or consequence of the low reliability.

A general point of concern is that the current MITI thresholds of proficiency are based upon expert opinion, and lack empirical support.^25^ This implies also that it is currently unclear which level of MI is minimally required to make a change.^33^

Subsequently, as delivering MI is more complex than generally believed even in the case of regular and intensive training,^34^ an even higher level of competency than currently stated would be necessary to reach significant effects in outcome.

An explanation for the mixed findings of inter-rater reliability could be that ratings of the first and second rater did not take place in the same time span, but 3 months later, which could have led to drift (decreased intra-rater variability and increased inter-rater reliability).^35^ On the other hand, the robustness of the MI fidelity findings during sensitivity analysis does not indicate this.

A comparison of the current findings with that of other intervention studies using MI is hampered by the frequent use of other instruments than the MITI^36^^–^38 or the usage of the previous version MITI 1.0 or 2.0 (e.g.,^39^).

In the MITI, the more important fidelity measures are either related to change talk or sustain talk or to a behavior change at a later stage.^20^ Therefore, important measures in the working chain of MI are (1) empathy and (2) spirit.^40^ Empathy, a fundamental factor in MI, although non-specific in psychotherapy, was associated with better client outcomes in MI delivered in the domain of addiction treatment.^20^ Spirit, which is a combination of the global scores autonomy/support, evocation, and collaboration, is a preliminary condition for change talk.^20^ Therein, the current finding of evocation is embedded.

In a study of Forsberg et al., it was shown that some counselors with monthly training sessions needed two and a half year to reach the level of beginning proficiency for the global variable spirit.^35^ In two other studies assessing MI training effects, the thresholds for spirit and empathy were exceeded immediately after the training.^41^^,^^42^

In the study presented, the reflections-to-questions ratio crossed the threshold for beginning proficiency and seemed also in another study easy to learn and cross.^35^ As a low amount of complex reflections was found in the current study, it can be concluded that it was difficult for the nurses to provide complex reflections. This was also mentioned in a study investigating MI skills and counselor characteristics before, during, and after MI training^39^ which is also in line with the statement that complex reflections are one of the hardest to improve skills during training.^22^^,^^43^^,^^44^

The current finding that MI non-adherent responses were less common in the MI-based intervention condition compared to the educational control condition is as promising as it is known that MI non-adherent counselor behaviors hinders the subsequent occurrence of change talk which predicts behavior change.^13^^,^^18^

Another explanation for the fact that not all MITI thresholds for beginning proficiency were crossed in the MI-based intervention condition could be due that the nurses had to follow a treatment manual since they participated in a scientific study. The usage of a manual could have resulted in a situation in which the counselor felt less free to completely focus on actual needs of the client (e.g. pushing too hard for commitment in line with the manual) resulting in a negative impact on the achieving sufficient effect sizes.^4^^,^^45^

Whereas for most of the MITI variables higher mean ratings of MITI sub scores in the MI-based intervention condition compared to the control condition were found, it could not be statistically discriminate between the two conditions for most of the MITI variables. This finding is in accordance with the study of Maissi et al. It has to be said that some of the before mentioned studies did not use the MITI to discriminate between conditions at all; thus, they were not included in this comparison.^15^^,^^43^^,^^44^ Only one study indeed provided differences in MITI scores between MI conditions.^41^

The current findings of differences in MI fidelity on nurse level in the intervention condition in the post-hoc analysis are in line with a study on MI training effects, which also showed a broad variation in counselor functioning^46^ and fluctuations over time.^35^

In addition, it seems that some counselors are not able to acquire skills^34^^,^^47^ whereas others may easily learn the new technique, no matter the extent of training provided. This inter-person difference is in line with findings of this study and also with some other studies in the field of the effects of MI-training.^35^^,^^48^^,^^49^ As a consequence, a stricter selection policy (e.g., for empathy) before entry as a potential solution for undesired variations in counselors’ MI functioning is advocated.^47^ In addition, future study results should enlighten the most effective MI training approaches as the important role of continuous supervision and feedback for MI practitioners is also reported elsewhere.^39^

Although in a systematic review of O’Halloran et al., higher results for the effectiveness of MI intervention were yielded if fidelity assessment had taken place,^12^ Lundahl et al. reported in contrast in their systematic review that checking MI fidelity was inversely related to MI outcomes.^5^ This was a surprising outcome, and those findings have to be seen in the light of two comments^50^^,^^51^ regarding methodological aspects of this review published. Apart from that, in accordance with Lundahl et al.,^5^ future studies are necessary and recommended to find an explanation for this phenomenon. The results of the underlying trial of this current study will be available next year and hopefully will shed some more light on this topic.

Although several studies investigating MI fidelity do not even mention reliability between two or more raters,^44^^,^^52^ results of the present and other studies (e.g.,^41^^,^^46^) seem to underscore that a study of rater reliability is required and the procedure has to include regular rater meetings to prevent rater drift and probably prevent to hamper reliability.^27^

At this moment, two overviews are available who describe a tool for treatment fidelity in health behavior change trials,^53^ and in trials using motivational interviewing specifically.^54^

Furthermore, several instruments are available for the assessment of MI quality. The MITI has shown to have good inter-rater reliability^27^ and predictive validity,^28^ and seems the most suitable if the specific focus is to specifically evaluate counselor behavior.

Furthermore, a limitation in the use of the MITI is that complex therapist (counselor) competence such as intentional or strategic use of MI may be insufficiently assessed^55^and one cannot evaluate the timing of interventions and techniques^56^ as well as that the MITI does not take into account the context in which an interview takes place. A second limitation is the very time consuming coding of the sessions by means of the MITI; intensive training of the raters and continuous consulting between raters on intervention-specific topics and MITI-specific rating topics is necessary in order to rate unanimously.

Some limitations of the present study need to be considered. First, the fidelity of the nurses in the MI intervention condition prior to or at the start of the trial was not assessed. This could have flawed the effectiveness of MI training and also the overall effectiveness of the MI intervention. Second, the first rater was not blinded for treatment allocation in the intervention versus control condition, which could have hampered validity of the findings. However, the finding that the blinded second rater confirmed the first rater’s findings invalidates this supposition.

## Conclusion

In general higher levels of MI fidelity in the intervention condition were found as well as were it possible to partially discriminate between MI-based intervention and education-based control condition. Although the quality of MI delivery as well as mixed inter-rater reliabilities of the fidelity scores leaves room for improvement, robust findings between the two raters and also their consensus approach were found.

Further analyses of the long-term effectiveness of the underlying trial will show whether a MI-based pre-treatment is more effective compared to a pain education pre-treatment and whether this improves participation and functioning of patients with fibromyalgia and chronic musculoskeletal pain undergoing pain rehabilitation.

## Implications for Behavioral Health

The present study confirms the need for rigor selection of MI counselors before training, and the important role of continuous supervision and feedback for MI practitioners in order to reach proper MI fidelity as well as the need for fidelity check in studies using MI.
